# Investigating the genetic causality between periodontitis and intracranial aneurysms: A 2-sample Mendelian randomization study

**DOI:** 10.1097/MD.0000000000046386

**Published:** 2025-11-28

**Authors:** Yao Chen, Jianhuang Huang, Quanming Zhou, Yuanbao Kang

**Affiliations:** a Department of Neurosurgery, Affiliated Hospital of Putian University, Putian, Fujian Province, China; b Department of Clinical Medicine, Fujian Medical University, Fuzhou, Fujian Province, China.

**Keywords:** causal relationship, intracranial aneurysms, Mendelian randomization, periodontitis

## Abstract

Previous studies have suggested an association between periodontitis and intracranial aneurysms (IAs). However, it remains unclear whether the association is causal. This study systematically investigated the potential genetic link between periodontitis and IAs, including the formation and rupture of IAs. This study utilized publicly available genome-wide association study summary statistics data for a 2-sample bidirectional Mendelian randomization (MR) analysis. The main statistical analysis method used was inverse variance weighting. The reliability of the results was verified through sensitivity analysis and assessment of the strength of genetic instrumental variables. There was no causal relationship between genetically determined periodontitis and uIAs (odds ratio [OR] = 1.02, 95% confidence interval [CI]: 0.89–1.17, *P* = .76) or aneurysmal subarachnoid hemorrhage (OR = 0.96, 95% CI: 0.85–1.08, *P* = .50). In the reverse MR analysis, the increased risk of uIAs or SAH was not statistically significant for an increased risk of periodontitis (uIAs: OR = 0.99, 95% CI: 0.94–1.05, *P* = .78; aneurysmal subarachnoid hemorrhage: OR = 1.02, 95% CI: 0.96–1.09, *P* = .51). The results from MR-Egger regression and weighted median method were consistent with the inverse variance weighted method. Sensitivity analysis indicated that horizontal pleiotropy was unlikely to distort the causal estimates. Our study does not support a causal relationship between periodontitis and the formation or rupture of IAs, and vice versa.

## 1. Introduction

Intracranial aneurysms (IAs) are severe cerebrovascular diseases characterized by pathological dilation of the intracranial artery wall. IAs are relatively common, occurring in 3 to 6 percent of the adult population.^[1]^ Clinically, IAs are present in unruptured intracranial aneurysms (uIAs) and aneurysmal subarachnoid hemorrhage (aSAH), which is a catastrophic complication with a mortality rate of 25% to 50%.^[2,3]^ Despite significant improvements in treatment methods and intensive care management, aSAH is still associated with substantial disability, death, and socioeconomic impact. Each year, approximately one-third of individuals with IAs die from aSAH; in half of survivors, additional support is required to continue normal daily activities.^[4]^ Furthermore, although the adoption of interventional techniques, novel materials, and treatment strategies have decreased mortality and disability rates in treating uIAs, the outcome is still not optimistic.^[5,6]^ Therefore, the mechanisms underlying the formation and progression of IAs, especially preventive and therapeutic measures, have garnered considerable attention from scholars.

Periodontitis, inflammation of the supporting tissues of the teeth, accompanied by a gradual loss of periodontal attachment and bone. Its characteristics include the formation of periodontal pockets and/or gingival recession, with an overall prevalence of 45% to 50%.^[7,8]^ Several recent observational studies have supported the association between periodontitis and IAs and suggested that periodontal bacteria may be involved in the pathology of IAs by spreading through gingival tissue into circulation.^[9–12]^ As such, periodontitis appears to be a modifiable nontraditional risk factor for IAs. However, this association is still lacking in validation from randomized controlled trials, and the evidence for a causal relationship is insufficient in strength.

Factors such as reverse causation and selection bias are inherent flaws in these observational studies. To address these limitations of observational studies, we employed Mendelian randomization (MR) analysis, using genetic variations as instrumental variables (IVs), to investigate the causal relationship between the exposure phenotype and the outcome phenotype.^[13]^ In this study, we performed a 2-sample MR analysis to assess the causal relationship between periodontitis and the risk of IAs formation and rupture. Additionally, we also performed reverse MR to investigate the causal relationship of IAs formation and rupture on periodontitis.

## 2. Material and methods

### 2.1. Data sources

The genome-wide association study (GWAS) summary statistics data used in this study for periodontitis were obtained from the FinnGen project (https://www.finngen.fi/en), led by the Finnish Biobank, which draws on longitudinal health registry data from the entire population of Finland since 1969. The GWAS summary data for periodontitis contained 3046 diagnosed cases and 1,95,395 control cases.

**Figure 1.**
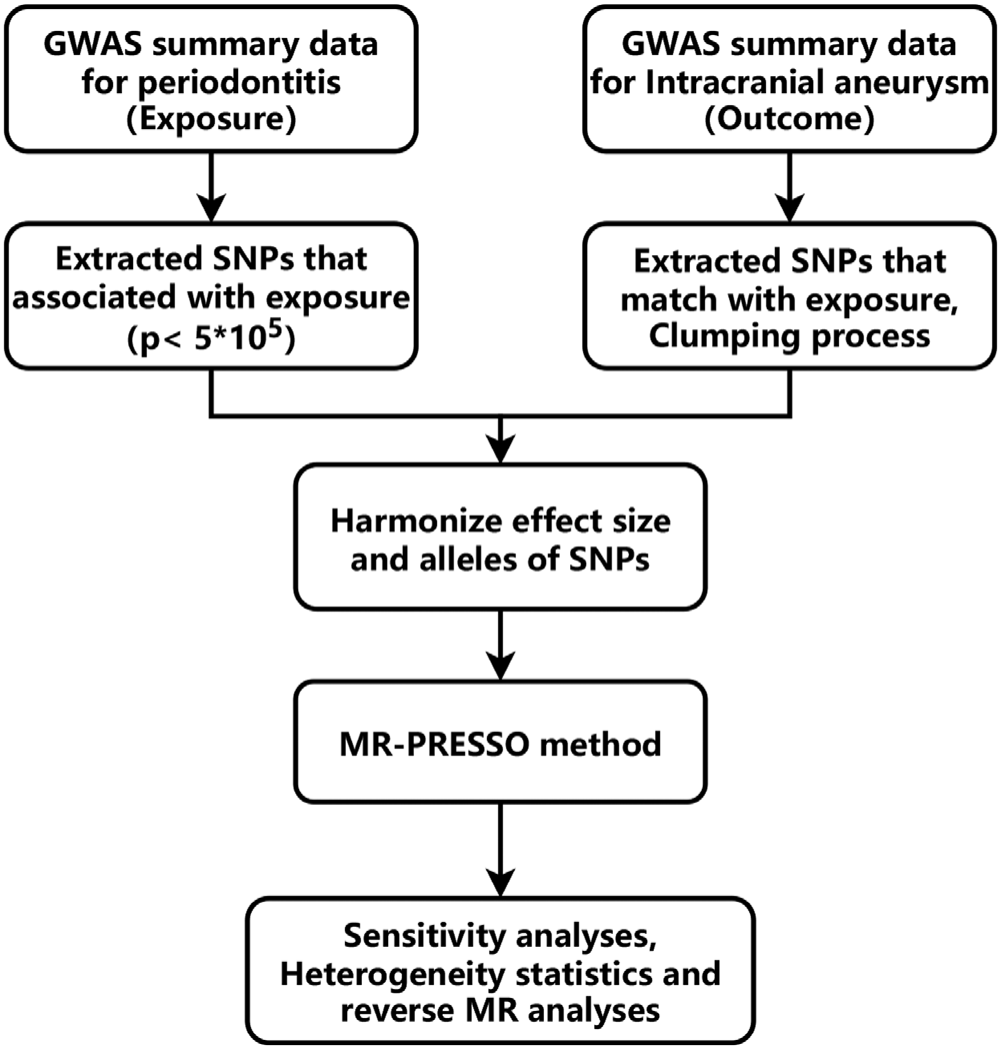
Flowchart of bidirectional Mendelian randomization analysis. GWAS = genome-wide association study, MR = Mendelian randomization, SNPs = single nucleotide polymorphisms.

The GWAS summary data for IAs were obtained from a meta-analysis conducted by Bakker^[14]^ et al, which included uIAs and aSAH cases. The former included 7495 diagnosed cases and 71,934 controls, while the latter included 5140 diagnosed cases and 71,952 control cases. To mitigate population stratification bias, all GWAS summary data were included only from individuals of European ancestry. The research workflow was illustrated in Figure 1.

### 2.2. Selection of IVs

To estimate causal effects using genetic instruments, it is essential to meet the 3 key assumptions of IVs. Therefore, quality control measures were implemented. First, since the number of independent single nucleotide polymorphisms (SNPs) with a strong association with periodontitis at *P* < 5 × 10^−8^ was limited, we set the threshold at *P* < 5 × 10^−5^ to select a sufficient number of IVs. Second, to exclude SNPs in strong linkage disequilibrium, the clumping was performed using default parameters. Third, SNPs with a minor allele frequency < 0.01 were eliminated. Fourth, the *F*-statistic for each SNP was calculated using the following equation: *F* = *R*^2^ × (N − 2)/(1 − *R*^2^). *R*^2^ represents the exposure variance for each instrumental variable interpretation. The filtering criterion was an *F*-test value > 10.^[15]^ Fifth, SNPs with allele inconsistency between exposure and outcome samples, as well as palindromic alleles were excluded. Lastly, the MR-PRESSO global test was applied to detect potential horizontal pleiotropy of SNPs, and SNPs with a *P* < .05 were excluded.^[16]^ Finally, we obtained a set of high-quality SNPs.

### 2.3. Statistical analysis

In this study, we employed multiple statistical analysis methods, including IVW, MR-Egger regression, and weighted median, to estimate the causal effects of exposure phenotypes on the outcome. The IVW method was the primary statistical approach used in this study. When all selected SNPs are valid IVs, the IVW method provides the most accurate results. MR-Egger regression method allows for consistent estimation of causal effects even in the presence of pleiotropy effects.^[17]^ The Weighted Median method is applicable to some or as many as 50% of SNPs that are invalid IVs and gives consistent estimates.^[18]^

### 2.4. Pleiotropic and sensitivity analysis

In this study, we employed various methods to detect the presence of pleiotropy in IVs. Firstly, the intercept term of MR-Egger regression can effectively indicate whether horizontal pleiotropy drives the results of the MR analysis.^[17]^ Secondly, the asymmetry of the funnel plot can reflect the presence of horizontal pleiotropy in IVs.^[19]^ Lastly, we conducted the MR-PRESSO test to assess the presence of pleiotropy in IVs.^[16]^ To identify heterogeneity in IVs, we used both the IVW and MR-Egger regression to quantify heterogeneity by Cochran’s *Q* statistic. Additionally, we performed leave-one-out analyses to examine the robustness and consistency of the MR analysis results.

In this study, the Bonferroni method was used to correct for *P*-values for multiple comparisons, that is, *P* < .006 (0.05/8) to show convincing evidence of causation. All analyses were conducted using the “TwoSampleMR”^[19]^ and “MRPRESSO”^[16]^ packages in R software version 4.3.1 (Vienna, Austria).

## 3. Results

### 3.1. Causal relationship of periodontitis with uIAs and SAH

**Figure 2.**
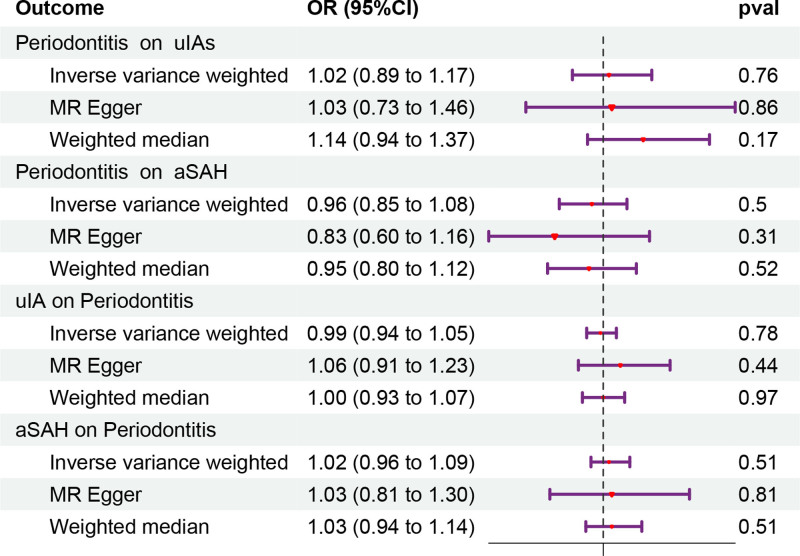
Mendelian randomization estimates of the causal relationship between periodontitis and uIAs/aSAH, and vice versa. The estimated values represent the odds ratio for each unit increase in the log odds of the exposure determined by the instrumental variable. The horizontal line indicates the 95% confidence interval. aSAH = aneurysmal subarachnoid hemorrhage, CI = confidence interval, MR = Mendelian randomization, OR = odds ratio, uIAs = unruptured intracranial aneurysms.

In the forward MR analysis, periodontitis was included as an exposure factor for uIAs and aSAH. A total of 18 independent SNPs with *P* < 5 × 10^−5^ were selected as IVs for uIAs, while 13 SNPs were selected for aSAH. The detailed information of these IVs can be found in Table S1, Supplemental Digital Content, https://links.lww.com/MD/Q846. These SNPs explained 2.13% and 2.72% of the variance for uIAs and aSAH, respectively. The *F*-statistic for each SNP was >10, indicating that the explained phenotype variance and high *F*-statistic confirmed the MR assumption (1). The MR estimates for the different methods were shown in Figure 2. The main results from the IVW method indicated that an increased risk of periodontitis was not statistically significant associated with an increased risk of uIAs (odds ratio [OR] = 1.02, 95% confidence interval [CI]: 0.89–1.17, *P* = .76) or aSAH (OR = 0.96, 95% CI: 0.85–1.08, *P* = .50). Additionally, the MR-Egger regression and weighted median method showed consistent negative results (Fig. 2).

### 3.2. Pleiotropic and sensitivity analysis

Although the exposure and outcome came from different samples, the *P*-values of the heterogeneity tests were all >.05, indicating no heterogeneity. The results of the MR-PRESSO global test and the horizontal pleiotropy test are not significant, suggesting that the IVs were only related to the exposure and not related to other confounding factors (Table 1). The funnel plot showed a roughly symmetric distribution of SNPs on both sides of the IVW method (Fig. 3). Low or no heterogeneity, no indication of potentially confounding SNPs, and pleiotropic robust analysis results ensured the validity of the MR assumptions (2) and (3). By performing leave-one-out analyses, no significant impact of individual SNPs on the test results was found, which reflects the robustness of the aforementioned conclusions (Tables S1 and S2, Supplemental Digital Content, https://links.lww.com/MD/Q846).

**Table 1:** Pleiotropic and sensitivity analysis in bidirectional MR* analysis.

Heterogeneity				
Exposure	Outcome	*Q* †	df‡	*P*val§
Periodontitis	uIAs‖	9.65	17.00	.92
Periodontitis	aSAH¶	9.90	12.00	.62
uIAs	Periodontitis	28.02	46.00	.98
aSAH	Periodontitis	35.95	48.00	.90

MR-PRESSO results				
Exposure	Outcome	Global.Test.RSSobs	Global.Test.*P*val	
Periodontitis	uIAs	11.00	0.90	
Periodontitis	aSAH	11.69	0.64	
uIAs	Periodontitis	29.32	1.00	
aSAH	Periodontitis	37.40	0.91	

MR-Egger regression for directional pleiotropy				
Exposure	Outcome	Intercept	SE#	*P*val
Periodontitis	uIAs	−0.01	0.03	.95
Periodontitis	aSAH	0.03	0.03	.40
uIAs	Periodontitis	−0.02	0.02	.35
aSAH	periodontitis	−0.01	0.02	.96

* MR = Mendelian randomization.

† *Q* = heterogeneity statistic *Q*.

‡ df = degree of freedom.

§ *P*val = *P*-value.

‖ uIAs = unruptured intracranial aneurysms.

¶ aSAH = aneurysmal subarachnoid hemorrhage.

# SE = standard error.

**Figure 3.**
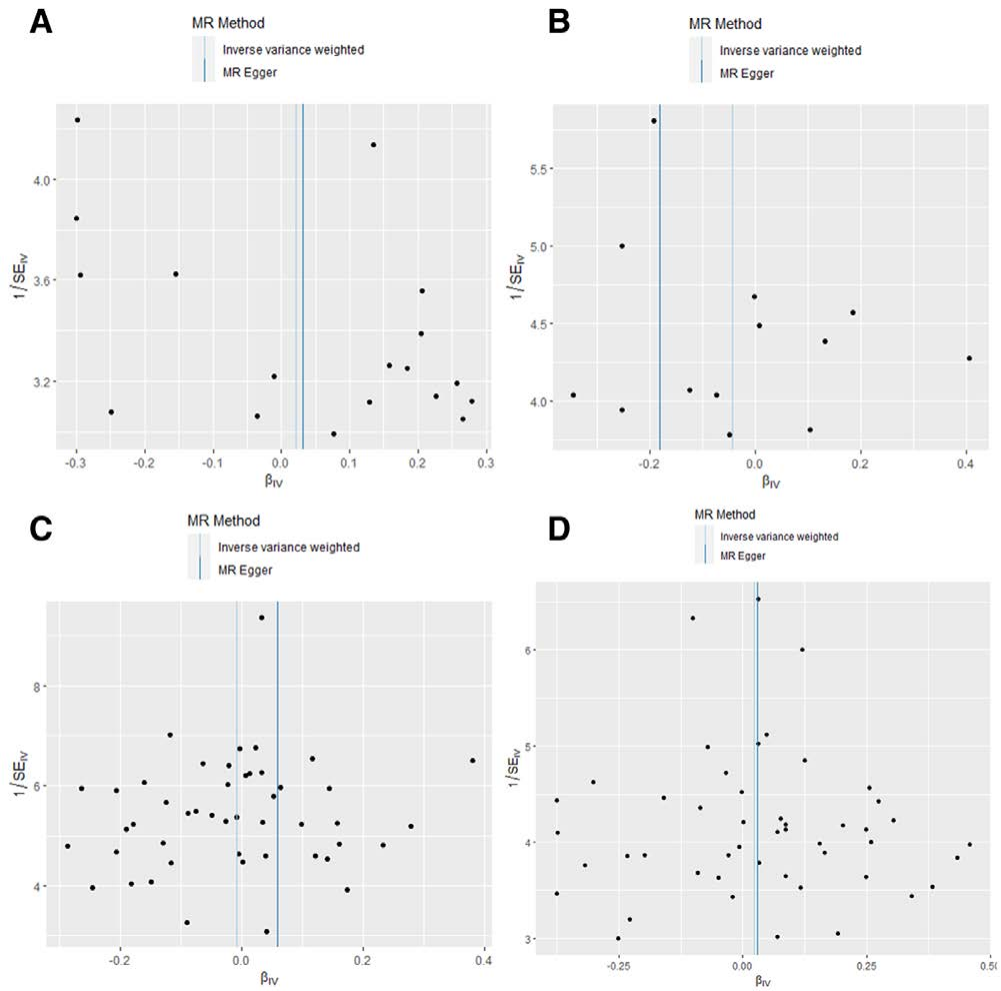
The funnel plot showed a roughly symmetric distribution of SNPs on both sides of the IVW method. (A) periodontitis on uIAs. (B) periodontitis on aSAH. (C) uIAs on periodontitis. (D) aSAH on periodontitis. aSAH = aneurysmal subarachnoid hemorrhage, IVW = inverse variance weighted, MR = Mendelian randomization, SNP = single nucleotide polymorphisms, uIAs = unruptured intracranial aneurysms.

### 3.3. Causal relationship of uIAs/SAH on periodontitis

Finally, this study also explored the causal relationship of uIAs/SAH on periodontitis. After screening, a total of 47 qualified IVs for uIAs and 49 qualified IVs for aSAH were obtained. The detailed information of IVs was listed in Table S2, Supplemental Digital Content, https://links.lww.com/MD/Q846. The primary results of IVW showed that there was no significant association between increased prevalence of uIAs or aSAH and increased risk of periodontitis (uIAs: OR = 0.99, 95% CI: 0.94–1.05, *P* = .78; aSAH: OR = 1.02, 95% CI: 0.96–1.09, *P* = .51). In addition, MR-Egger regression and weighted median showed consistent results (Fig. 2). The *P*-values of the heterogeneity tests, MR-PRESSO global test, and horizontal pleiotropy test were all >.05 (Table 1), indicating the robustness of this conclusion.

## 4. Discussion

This study utilized the genetic correlation of SNPs to investigate the association between periodontitis and IAs, including their formation or rupture. Our 2-sample MR analysis did not observe a causal relationship between periodontitis occurrence and the formation or rupture of IAs in the European population sample. Similarly, the reverse MR analysis also found no evidence of an association between the formation or rupture of IAs and periodontitis. To the best of our knowledge, this is the first study to explore the bidirectional causal relationship between periodontitis and the formation or rupture of IAs through the MR method.

Previous epidemiological research has suggested a potential association between periodontitis and the formation of IAs. A case-control study found a significant correlation between poor periodontal health and the formation of IAs.^[20]^ A cohort study revealed an association between periodontitis and the formation of IAs (OR: 5.99, 95% CI: 2.6–13.8, *P* < .001).^[9]^ Another large-scale cohort study conducted in Korea also showed a significant correlation between the presence of periodontitis and an increased risk of IAs (HR: 1.21, 95% CI: 1.09–1.34, *P* < .001).^[21]^ Furthermore, there may be a potential association between periodontitis and aSAH. In a 14-year follow-up study of periodontitis patients, baseline periodontitis increased the risk of aSAH (HR: 14.3, 95% CI: 1.5–135.9, *P* = .020).^[9]^ Additionally, the KUH cohort demonstrated that periodontitis was associated with an increased risk of aSAH diagnosis during the follow-up period (OR: 5.3, 95% CI: 1.1–25.9, *P* < .000).^[10]^ Despite the relatively consistent findings, it is still challenging to establish a causal relationship between periodontitis and the formation or rupture of IAs due to limitations of observational studies, such as selection bias and confounding factors.

The study conducted a bidirectional MR analysis, which resulted in consistent nonsignificant findings. The OR values for both hypotheses were slightly above and below 1, with highly overlapping CIs. This indicates a weak clinical significance of the observed effects. MR analysis typically provides strong evidence in situations where the effect size is particularly small or nonexistent.^[22]^ Meanwhile, our stringent instrumental variable selection protocol, along with the MR-PRESSO global test and horizontal pleiotropy assessment, ensures the robustness of the MR analysis results. Therefore, it is unlikely that there is any causal relationship between periodontitis and IAs formation or rupture. Although our MR analysis did not reveal a causal relationship between periodontitis and IAs, previous observational studies have suggested potential mechanistic links between the 2 conditions. Periodontitis – a chronic inflammatory disease triggered by bacterial pathogens – may allow microbial products from inflamed gingival tissues to enter the bloodstream. Theoretically, this could contribute to systemic inflammation, vascular endothelial dysfunction, or vascular wall remodeling, processes implicated in aneurysm formation and rupture.^[9–12]^ For instance, inflammatory mediators associated with periodontitis (e.g., IL-1β, TNF-α) may enhance matrix metalloproteinase activity, potentially weakening arterial walls. However, our findings indicate that these mechanisms may not translate into direct causal effects, possibly due to compensatory physiological processes or confounding by factors such as smoking and hypertension.^[14,23,24]^ Meanwhile, we adopt a rigorous IVs screening program, MR-PRESSO global test, and horizontal pleiotropy tests ensured the robustness of the MR analysis results. However, our study has several limitations. First, the generalizability of our findings is restricted to populations of European ancestry Second, although the IVs we used for periodontitis and IAs were sufficiently robust to avoid. major weak instrument bias (*F*-statistic > 10), the proportion of variance explained by SNPs for periodontitis was limited (e.g., ~2.1–2.7% in the forward analysis). This implies that our statistical power to detect very small causal effects (ORs very close to 1) was constrained. Consequently, we cannot entirely rule out the possibility of extremely small causal effects of periodontitis on IAs (or vice versa) that fall below the detection threshold of our current analysis. Nevertheless, the observed effect estimates (ORs ranging from 0.96 to 1.02) were extremely close to the null value of 1.0, and their CIs effectively excluded moderate or large effects. Thus, while acknowledging this power limitation, our results robustly suggest the absence of clinically relevant causal effects. Additionally, considering the intermediate position of MR analysis in the evidence pyramid, future randomized controlled trials may still be worth considering.^[25]^

## 5. Conclusions

In conclusion, our study does not support a causal relationship between periodontitis and the formation or rupture of IAs, nor vice versa. Further randomized controlled trials are needed to validate these findings and obtain more reliable and conclusive results.

## Acknowledgments

The authors acknowledge and thank the investigators of the original GWAS studies for sharing summary data used in this study.

## Author contributions

**Data curation:** Quanming Zhou, Yuanbao Kang.

**Investigation:** Jianhuang Huang, Yuanbao Kang.

**Methodology:** Jianhuang Huang.

**Software:** Jianhuang Huang.

**Writing – original draft:** Yao Chen.

**Writing – review & editing:** Yao Chen, Jianhuang Huang.

## Supplementary Material


